# Intensive Care Unit Acquired Weakness Is Associated with Rapid Changes to Skeletal Muscle Proteostasis

**DOI:** 10.3390/cells11244005

**Published:** 2022-12-11

**Authors:** Mustafa Ozdemir, Matthew P. Bomkamp, Hayden W. Hyatt, Ashley J. Smuder, Scott K. Powers

**Affiliations:** 1 Department of Applied Physiology and Kinesiology, University of Florida, Gainesville, FL 32611, USA; 2 Department of Internal Medicine, Division of Cardiovascular Health and Disease, University of Cincinnati, Cincinnati, OH 45267, USA; 3 Department of Physiology, The Johns Hopkins University School of Medicine, Baltimore, MD 21201, USA; 4 Department of Health Sciences, Stetson University, Deland, FL 32720, USA

**Keywords:** mechanical ventilation, respiratory muscles, limb muscles, protein synthesis, degradation, anabolic signaling

## Abstract

Intensive care unit (ICU)-acquired weakness is a frequent consequence of critical illness that impacts both the limb and respiratory muscles. The cause of ICU-acquired weakness is multifactorial, but both prolonged limb muscle inactivity and mechanical ventilation are risk factors for muscle wasting, which predisposes ICU patients to both short-term complications and long-term disabilities resulting from muscle weakness. Unfortunately, the current research does not provide a detailed understanding of the cellular etiology of ICU-acquired weakness, and no standard treatment exists. Therefore, improving knowledge of the mechanisms promoting muscle atrophy in critically ill patients is essential to developing therapeutic strategies to protect against ICU-induced skeletal muscle wasting. To advance our understanding of the mechanism(s) responsible for ICU-acquired weakness, we tested the hypothesis that ICU-induced muscle inactivity promotes a rapid decrease in anabolic signaling/protein synthesis and accelerates proteolysis in both limb and respiratory muscles. To investigate ICU-induced changes in skeletal muscle proteostasis, adult Sprague Dawley rats were anesthetized and mechanically ventilated for 12 h to simulate ICU care. Measurements of anabolic signaling, protein synthesis, and proteolytic activity in the limb muscles (plantaris and soleus) and respiratory muscles (parasternal and intercostal) revealed ICU-induced reductions in both anabolic signaling (i.e., AKT/mTOR pathway) and muscle protein synthesis. Moreover, simulated ICU care resulted in increased biomarkers of accelerated proteolysis in both limb and respiratory muscles. These novel findings reveal that disturbances in limb and respiratory muscle proteostasis occur rapidly during ICU-induced muscle inactivity, irrespective of the muscle function or muscle fiber type.

## 1. Introduction

Critical care medicine provides life-saving treatments to millions of people annually. However, a common and unintended consequence of prolonged intensive care is the development of skeletal muscle myopathy, which often develops secondary to the condition necessitating intensive care unit (ICU) admission [1,2]. Precisely, ICU-acquired weakness is a clinical condition characterized by limb and/or respiratory muscle wasting, occurring due to no plausible etiology other than critical illness and bed rest [2,3,4]. Prolonged disuse of the limb muscles during bed rest and the inactivity of respiratory muscles in patients requiring ventilatory support are postulated to be key contributors to ICU-acquired weakness. 

It is established that mechanical ventilation (MV)-induced diaphragm inactivity results in diaphragm fiber atrophy and weakness due to both accelerated proteolysis and reduced protein synthesis [5,6,7,8,9]. This condition is referred to as ventilator-induced diaphragm dysfunction (VIDD); importantly, VIDD is likely a contributor to the inability to wean patients from MV [8]. In contrast to the extensive knowledge about VIDD, limited information exists regarding the impact of ICU-acquired weakness on proteolysis and the rates of protein synthesis in accessory respiratory muscles and locomotor muscles with different fiber types. Improving our understanding of ICU-acquired weakness is important because the development of ICU-acquired weakness is independently associated with higher mortality and lower physiological functions at 6 months following ICU discharge [10]. Moreover, recent clinical findings suggest that both respiratory and limb muscle weakness are independent predictors of the need for prolonged MV, and that limb muscle weakness is associated with greater rates of weaning failure from the ventilator [11]. Additionally, ICU-acquired weakness can promote long-term deleterious consequences to overall health and quality of life by increasing the duration of hospitalization, impairing physical mobility, and prolonging rehabilitation following hospital discharge [2,3,11,12]. 

Although several interventions to protect against ICU-acquired weakness have been proposed (i.e., exercise, neuromuscular electrical stimulation, nutritional support, etc.), no standard therapy for the prevention of ICU-acquired weakness exists [11]. Therefore, an improved understanding of the mechanisms that contribute to respiratory and locomotor muscle ICU-acquired weakness is essential to developing effective therapeutic countermeasures. To advance our understanding of ICU-acquired weakness, this preclinical study investigated the impact of ICU care on proteostasis in both limb and respiratory muscles during the first 12 h of simulated ICU treatment. Specifically, we tested the hypothesis that ICU-induced muscle inactivity results in a rapid decline in anabolic signaling/protein synthesis and accelerates proteolysis in both the limb and respiratory muscles. Our results support this hypothesis and provide the first evidence that disturbances in proteostasis develop rapidly in both locomotor and respiratory muscles during the first 12 h of a simulated ICU environment that includes MV.

## 2. Materials and Methods

### 2.1. Animals, Institutional Approval and Experimental Design

**Figure 1:**
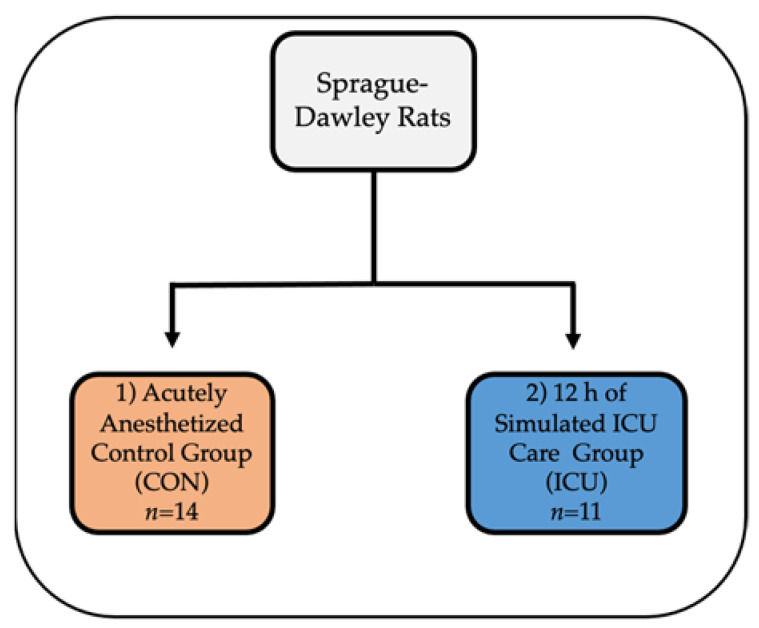
Experimental design. Two groups. (1) Acutely anesthetized control group (CON, *n* = 14). *N* = 14. (2) 12 h of simulated ICU care group (ICU, *n* =11).

Young adult (~4–6 months old) female Sprague Dawley rats were randomly assigned to one of two experimental groups: (1) acutely anesthetized control group (CON, *n* = 14), and (2) ICU simulation group (ICU, *n* = 11) (Figure 1). Prior to the experiments, the animals were housed in a 12:12 h light cycle with food and water provided ad libitum. Animals were allowed to acclimate in the animal housing facility for 7 days prior to experimentation after being purchased from an accredited vendor. The animals were cared for in accordance with the Guide for the Care and Use of Laboratory Animals. These experiments were approved by the Institutional Animal Care and Use Committee of the University of Florida.

No differences in body weight existed between the two groups at the start of the experiment (CON = 291 g ± 7.2; ICU = 286 g ± 3.2). CON animals remained awake and ambulatory in their cages throughout the 12 h experimental time period. At the endpoint, CON animals were acutely anesthetized using intraperitoneal (IP) injections with sodium pentobarbital (60 mg/kg body weight). Upon reaching a surgical plane of anesthesia, the animals received an IP injection of puromycin (0.04 µmol/g of body weight) (InvivoGen, San Diego, CA, USA) for the measurement of protein synthesis [13]. The incorporation of puromycin into growing polypeptide chains (i.e., protein synthesis rate) was later determined by immunoblotting for puromycin in the total protein extracts via Western blotting. Thirty minutes following the injection of puromycin, the animals were euthanized, and the soleus, plantaris, parasternal intercostal, and intercostal (intact external and internal intercostal) muscles were promptly removed. Tissues were rapidly frozen in liquid nitrogen and stored at −80 °C for subsequent biochemical analysis. A section from each muscle was rapidly frozen in an OCT medium at an unstressed length in isopentane and was chilled to the temperature of liquid nitrogen. These samples were then stored at −80 °C for the subsequent measurement of the muscle fiber cross-sectional area (CSA). Animals in the ICU group were anesthetized using IP injections with sodium pentobarbital (60 mg/kg body weight) and underwent 12 h of controlled MV. Throughout the 12 h MV period, the animals remained immobile with their limbs passively stretched every two hours. Animals received puromycin (0.04 µmol/g of body weight) identical to the CON group thirty minutes prior to the end of the 12 h experimental period and were euthanized thirty minutes later. Tissues were then removed and stored identically in the CON group.

### 2.2. Simulated ICU Protocol

All surgical procedures incorporated aseptic techniques. Briefly, the animals received an IP injection of sodium pentobarbital (60 mg/kg body weight). After reaching a surgical plane of anesthesia, animals underwent a tracheostomy and were mechanically ventilated for 12 h with a pressure-controlled ventilator that provided the full work of breathing (Servo Ventilator 300; Siemens, Munich, Germany). Ventilator settings included a respiratory rate of 80/breaths per minute with the positive end-expiratory pressure established at 1 cm H_2_O. Following the tracheostomy, the carotid artery and jugular vein were cannulated for the measurement of arterial blood pressure and the continuous infusion of anesthesia (sodium pentobarbital, ~10 mg/kg/hr) during the experiments. Arterial blood samples were obtained periodically to monitor arterial blood gas values (PaCO_2_, PaO_2_, and pH) (GEM Premier3000; Instrumentation Laboratory, Lexington, MA, USA). Adjustments to the ventilator settings were performed to control alveolar ventilation and fraction of inspired oxygen to ensure the maintenance of blood gas and pH homeostasis during ventilator support. Throughout the 12 h of MV, the PaO_2_ was maintained >60 mmHg, and PaCO_2_ was sustained <40 mmHg. Continuous care of the animals was provided during MV in the form of eye lubrication, expressing the bladder, removing mucus from the airway, passive limb movement, and rotating the animals’ position to combat blood pooling. Animal body temperature was maintained at ~37 °C using a recirculating warming blanket. Additionally, the animals received glycopyrrolate (0.02 mg/kg) intramuscularly every 2 h to reduce airway secretions.

### 2.3. Skeletal Muscle Fiber Cross-Sectional Area

Frozen muscle sections were cut at a thickness of 10 µm with a cryostat (HM 550 Cryostat, Thermo Fisher, Waltham, MA, USA) and were stained for dystrophin (Thermo Fisher, #RB-9024-R7) myosin heavy chain I (Hybridoma Bank, A4.840s IgM 1:15, Developmental Studies, Iowa City, IA, USA) and myosin heavy chain IIa (Hybridoma Bank, SC-71c IgG 1:50) for the analysis of CSA. After identifying type I and IIa fibers, the remaining muscle fibers were classified as type IIx/IIb fibers. Images were acquired via a monochrome camera attached to an inverted fluorescent microscope with 10× magnification (Axiovert 200, Zeiss, Germany). The muscle fiber type and CSA were analyzed using Image J software (National Institutes of Health, Bethesda, MD, USA).

### 2.4. Western Blot Analysis

Muscle samples were homogenized 1:10 (mg wt/µL buffer) in 5 mM Tris-HCL with 5 mM EDTA at a pH of 7.4 with protease and phosphatase inhibitor cocktail (Millipore Sigma, St. Louis, MO, USA). Skeletal muscle homogenates were centrifuged at 1500× *g* for 10 min at 4 °C. The supernatant was separated, and the protein concentration was assessed using the Bradford method (Millipore Sigma), followed by the normalization of protein concentrations in 2× Laemmli sample buffer (#1610737, Bio Rad, Hercules, CA, USA) with 5% (w/vol) dithiothreitol which was boiled at 100 °C for 5 min. Samples were loaded on 4–20% gradient Criterion TGX gels (Bio-Rad), separated by electrophoresis, and then transferred to LF-PVDF membrane (Millipore Sigma). Following the transfer, membranes were blocked in a 5% non-fat milk solution in PBS for 1 h at room temperature. Membranes were then incubated with the primary antibody of interest overnight at 4 °C. Following incubation of the secondary antibodies for 1 h at room temperature, membranes were scanned and analyzed with a Li-Cor Odyssey Infrared Imager (Li-Cor Biosciences, Lincoln, NE, USA) using Odyssey 2.1 software (Li-Cor Biosciences, Lincoln, NE, USA). Primary antibodies of interest were anti-puromycin (#EQ0001) (Kerafast, Boston, MA, USA), anti-mTOR (#2983), anti-p-mTOR (#5536), anti-AKT (#2938), anti-p-AKT (#9271), anti-4E-BP1 (#9644), anti-p-4E-BP1 (#2855), anti-STAT3 (#4904), anti-p-STAT3 (#9145), anti-LC3 (#2775) (Cell Signaling Technology, Danvers, MA, USA), 4-hydroxynonenal (4-HNE) (Abcam, Cambridge, MA, USA, ab46545) and anti-alpha II spectrin (sc-48382) (Santa Cruz, Dallas, TX, USA). The membranes were exposed to either IRDye 680RD or 800CW goat anti-rabbit or mouse IgG secondary antibody (1:10,000; Li-Cor Biosciences) for 1 h at 25 °C. All protein concentrations were normalized to the total protein using Revert Total Protein (Li-Cor Biosciences Lincoln, NE, USA). Membranes blotted for phosphorylated proteins were subsequently stripped using LI-COR NewBlot stripping buffer (LI-COR Biosciences) and probed for the respective total target protein.

### 2.5. Real-Time Polymerase Chain Reaction

The total RNA was isolated from each muscle of interest with the TRIzol reagent (Invitrogen, Carlsbad, CA, USA). RNA concentration was determined by spectrophotometry, and 5 µg was reverse transcribed using the Superscript III First-Strand Synthesis System (Thermo Fisher, Waltham, MA, USA) according to the manufacturer’s protocol. One µL of cDNA was added to a 24 µL master mix containing nuclease-free water (Millipore Sigma), Taqman master mix (Thermo Fisher), and assay primer. The gene expression was quantified using the 2^−ΔΔ*CT*^ method. MAFbx/Atrogin-1 (Rn00591730_m1), MuRF1 (Rn00590197_m1), and β-Glucuronidase (reference gene; GUSB, Rn00667869_m1) transcripts were assayed using inventoried rat primer and probe sequences commercially available (Thermo Fisher).

### 2.6. Statistical Analysis

Data for each dependent measure were analyzed using a Shapiro–Wilk test to determine if the data were normally distributed. An independent student’s *t*-test was conducted for the data sets with a normal distribution. By contrast, when the data were not normally distributed, a Mann–Whitney U test was used to determine if the groups were statistically different. Significance was established at *p* < 0.05. Data are expressed as mean ± standard error of the mean. All analyses were performed using a commercial statistical analysis package (Prism, version 8.0; GraphPad Software, San Diego, CA, USA).

## 3. Results

### 3.1. Simulated ICU Care Depresses Skeletal Muscle Protein Synthesis and Signaling

**Figure 2:**
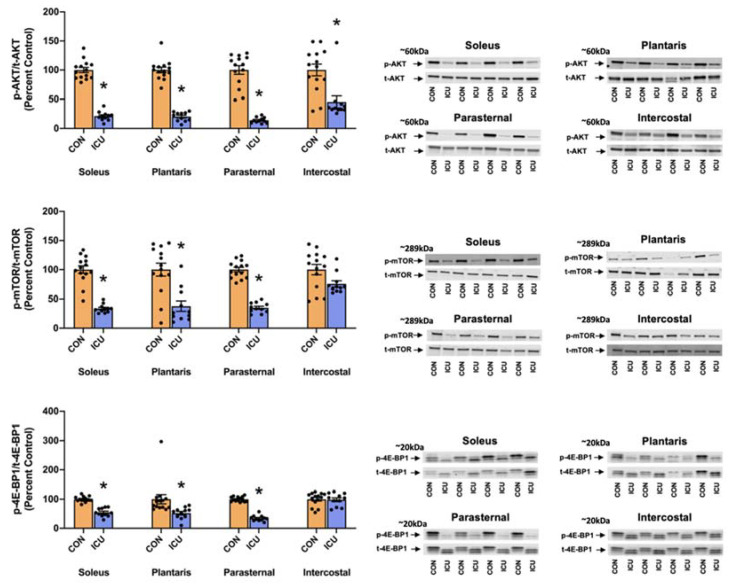
Muscle protein synthesis signaling. Ratio of p-AKT/t-AKT (Ser 473), p-mTOR/t-mTOR (Ser 2448) and p-4E-BP1/t-4E-BP1 (Thr 37/46) in the soleus, plantaris, parasternal, and intercostal muscles of control, non-anesthetized, non-ventilated rats (CON), and rats that underwent 12 h of controlled mechanical ventilation (ICU). Representative Western blots are shown to the right of the graph. Data are presented as mean ± SEM. * significantly different vs. CON (*p* < 0.05).

**Figure 3:**
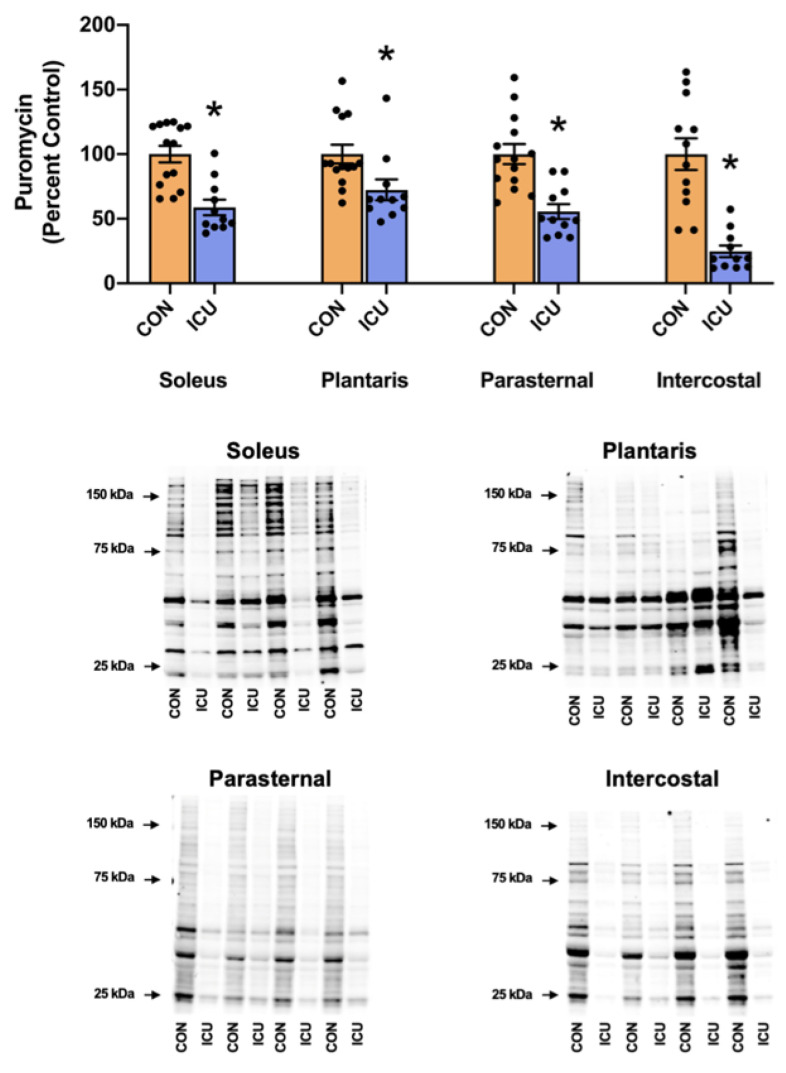
Muscle protein synthesis. Protein expression of puromycin incorporation into the soleus, plantaris, parasternal, and intercostal muscles of control, non-anesthetized, non-ventilated rats (CON), and rats that underwent 12 h of controlled mechanical ventilation (ICU). Representative Western blots are shown below the graph. All protein concentrations were normalized to total protein. Data are presented as mean ± SEM. * significantly different vs. CON (*p* < 0.05).

We hypothesized that as few as 12 h of ICU-induced muscle inactivity promoted disturbances in proteostasis within limb and respiratory muscles due to depressed anabolic signaling/protein synthesis and accelerated proteolysis. To test this hypothesis, we measured indicators of anabolic signaling/muscle protein synthesis and biomarkers of proteolysis. As hypothesized, simulated ICU care resulted in the decreased abundance of three biomarkers of anabolic signaling (i.e., phosphorylation of AKT, mTOR, and 4E-BP1) in both the soleus and plantaris muscles (Figure 2). Moreover, ICU care resulted in significant decreases in protein synthesis in both the soleus and plantaris muscles (Figure 3). 

Similarly, following 12 h of prolonged MV and simulated ICU care, the three biomarkers of anabolic signaling decreased in the parasternal intercostal muscles (Figure 2). Although prolonged MV also decreased phosphorylated AKT in the intercostal muscles, the phosphorylation status of both mTOR and 4E-BP1 was not significantly lower than the control. Nonetheless, protein synthesis was significantly depressed in both the parasternal intercostal and intercostal muscles (Figure 3). 

### 3.2. Simulated ICU Care Rapidly Increases Biomarkers of Skeletal Muscle Proteolysis

**Figure 4:**
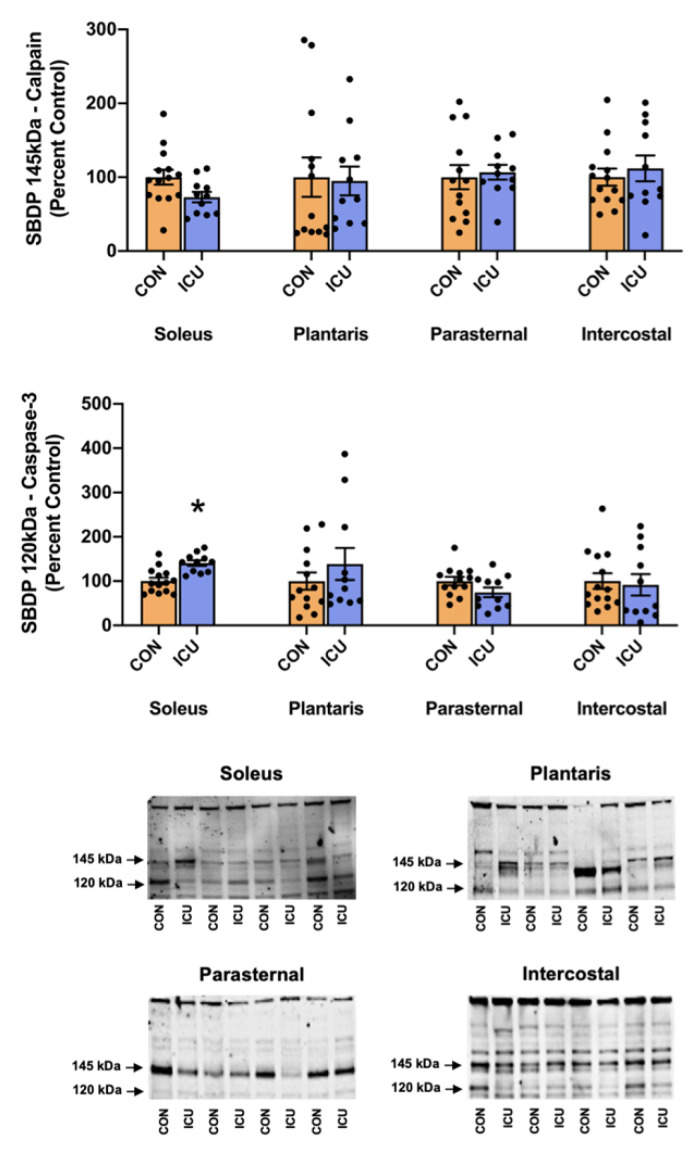
Calpain and Caspase-3 activity. 145 kDa spectrin breakdown product (SBDP) (calpain-specific cleavage) and 120 kDa SBDP (caspase-3-specific cleavage) in the soleus, plantaris, parasternal, and intercostal muscles of control, non-anesthetized, non-ventilated rats (CON), and rats that underwent 12 h of controlled mechanical ventilation (ICU). Representative Western blots are shown below the graph. Data are presented as mean ± SEM. * significantly different vs. CON (*p* < 0.05).

Skeletal muscle protein degradation was assessed by the measurement of several key markers of proteolytic activity. To evaluate the impact of simulated ICU care on the activation of calpains and caspase-3, we measured two well-established biomarkers of calpain and caspase-3 activity. Specifically, the cytoskeletal protein αII-spectrin is cleaved into two different signature fragments by active calpain and caspase-3 proteases; the 145-kDa spectrin breakdown product (SBDP) is indicative of calpain activity, and the 120-kDa SBDP is a sensitive biomarker of caspase-3 activation [14,15]. Our findings reveal that 12 h of simulated ICU care does not activate calpain in the limb or respiratory muscles (Figure 4). Similarly, 12 h of simulated ICU care does not activate caspase-3 activity in the plantaris, parasternal intercostal, and intercostal muscles. However, 12 h of ICU care does activate caspase-3 in the soleus muscle (Figure 4).

**Figure 5:**
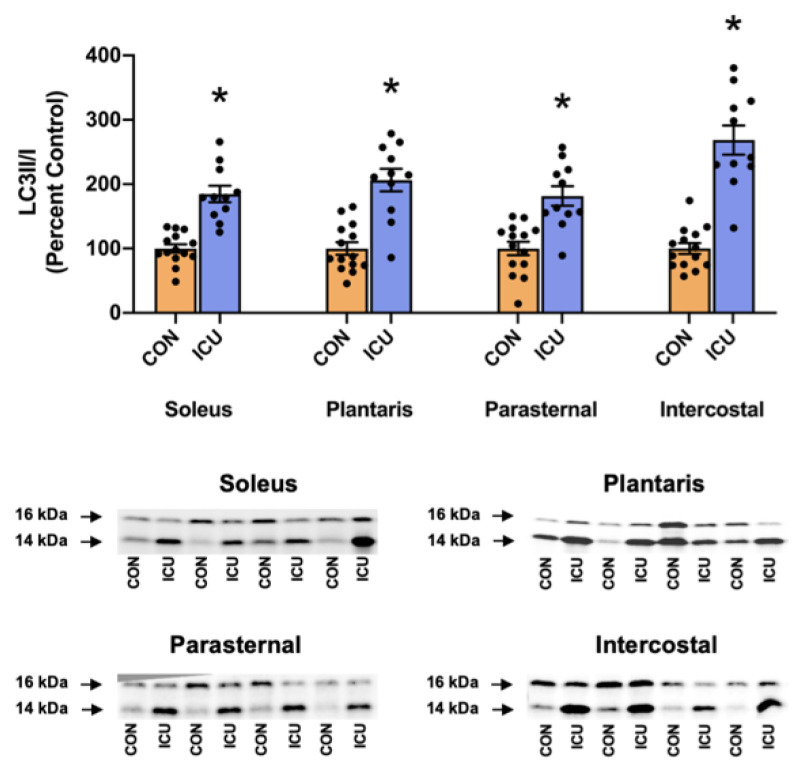
Ratio of LC3II/I as a marker of autophagy signaling. Ratio of LC3II/I in the soleus, plantaris, parasternal, and intercostal muscles of control, non-anesthetized, non-ventilated rats (CON), and rats that underwent 12 h of controlled mechanical ventilation (ICU). Representative Western blots are shown below the graph. Data are presented as mean ± SEM. * significantly different versus CON (*p* < 0.05).

Protein degradation by the autophagy/lysosome system was evaluated by the ratio of LC3II/I. During autophagy, LC3II is produced following the conjugation of LC3I with phosphatidylethanolamine (PE) on the surface of nascent autophagosomes and serves as an index of autophagosome formation [16]. Our results disclose that, compared to the control, the LC3II/I ratio was significantly increased in both limbs and the respiratory muscles of the animals exposed to 12 h of simulated ICU care (Figure 5).

**Figure 6:**
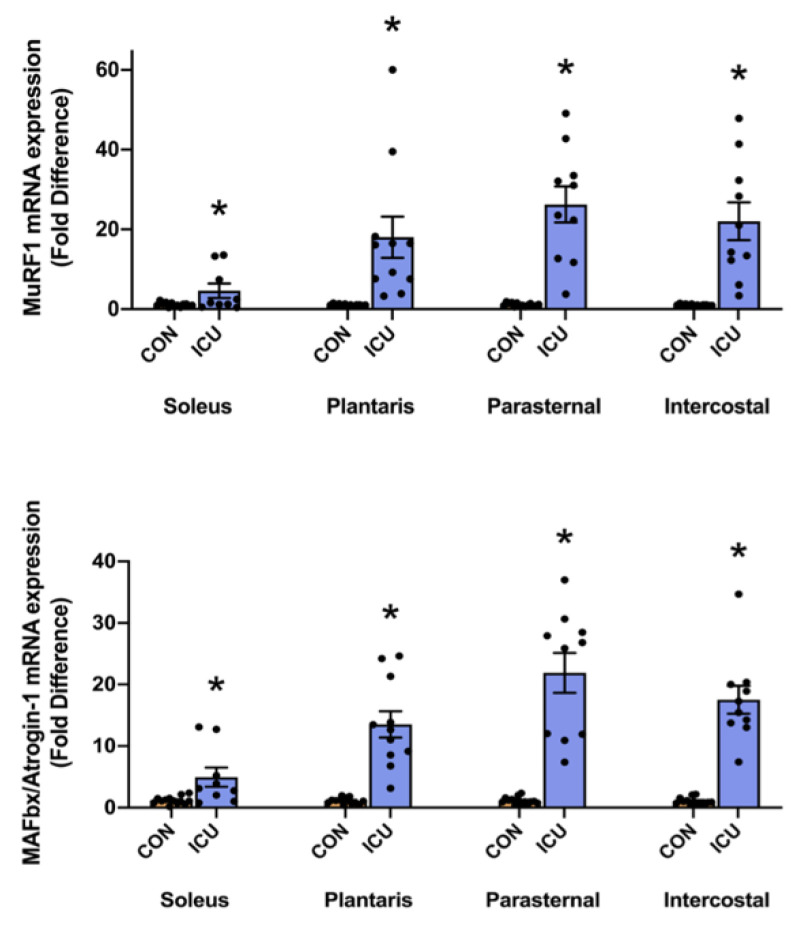
Ubiquitin-proteasome system. mRNA levels of the E3 ligases MuRF1 and MAFbx/Atrogin-1 in the soleus, plantaris, parasternal, and intercostal muscles of control, non-anesthetized, non-ventilated rats (CON), and rats that underwent 12 h of controlled mechanical ventilation (ICU). Data are presented as mean ± SEM. * significantly different versus CON (*p* < 0.05).

Finally, mRNA levels of the muscle-specific E3 ligases MuRF1 and MAFbx/Atrogin-1 were evaluated as biomarkers of ubiquitin-proteasome system signaling. Compared to the control, mRNA levels of both MuRF1 and MAFbx/Atrogin-1 were significantly increased in the hindlimb and respiratory muscles of animals exposed to 12 h of a simulated ICU environment (Figure 6).

### 3.3. ICU-Induced Activation of STAT3 

A hallmark of MV-induced diaphragmatic atrophy is the activation of signal transducer and activator of transcription 3 (STAT3) in diaphragm muscle fibers. As an active transcriptional activator, phosphorylated STAT3 can negatively impact mitochondrial function by altering the gene expression of Bim, UCP, and Cox5a and reducing membrane potential, and/or decreasing the efficiency of ATP generation [17]. Moreover, phosphorylated STAT3 can directly impact mitochondrial function by increasing mitochondrial ROS production and oxidative stress [17]. Therefore, to determine if STAT3 signaling is activated in the limb and respiratory muscles during ICU care, we measured the levels of phosphorylated STAT3. In addition, we measured the levels of 4-HNE conjugated proteins as a biomarker of oxidative stress.

**Figure 7:**
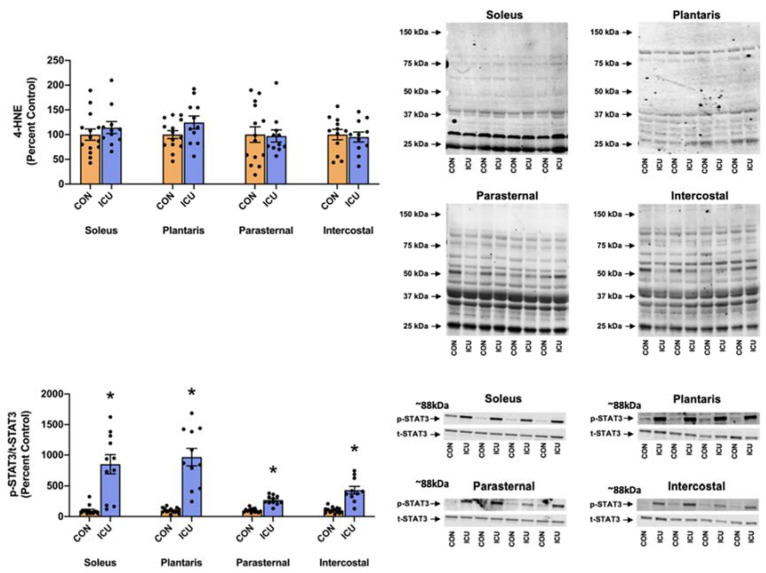
Stress signaling. 4-Hydroxynonenal (4-HNE) and p-STAT3/t-STAT3 (Tyr 705) in the soleus, plantaris, parasternal, and intercostal muscles of control, non-anesthetized, non-ventilated rats (CON), and rats that underwent 12 h of controlled mechanical ventilation (ICU). Representative Western blots are shown to the right of the graph. Data are presented as mean ± SEM. * significantly different vs. CON (*p* < 0.05).

Importantly, 12 h of simulated ICU care resulted in significant increases in phosphorylated STAT3 in both limb muscles (soleus and plantaris) and respiratory muscles (parasternal intercostal and intercostal) (Figure 7). Specifically, our findings reveal that the ratio of phosphorylated to total STAT3 protein was significantly elevated in the soleus, plantaris, parasternal intercostal, and intercostal muscles (Figure 7). By contrast, no differences existed between the 4-HNE levels in the limb muscles and respiratory muscles between the experimental groups (Figure 7).

### 3.4. ICU-Induced Muscle Fiber Atrophy

**Figure 8:**
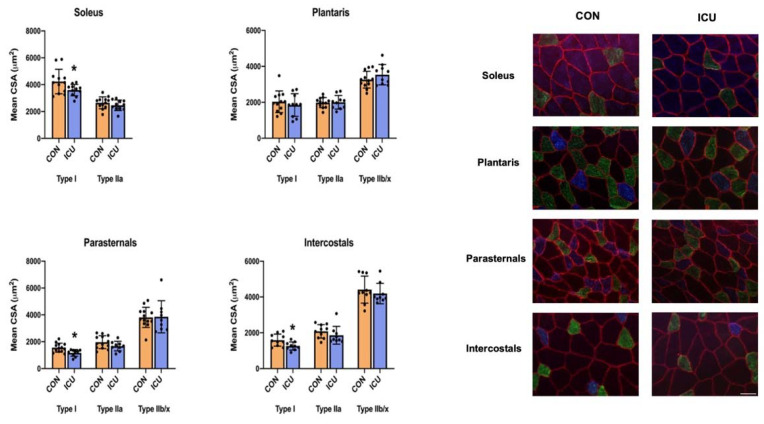
Muscle fiber cross-sectional area. Cross-sectional area of fiber types of the soleus, plantaris, parasternal, and intercostal muscles of control, non-anesthetized, non-ventilated rats (CON), and rats that underwent 12 h of controlled mechanical ventilation (ICU). Data are presented as mean ± SEM. * significantly different vs. CON (*p* < 0.05).

As the decisive measure of ICU-disturbed proteostasis within the limb and respiratory muscles, we measured the CSA of all muscle fiber types. Following 12 h of simulated ICU care, the CSA of type I muscle fibers was significantly reduced in both the respiratory muscles (parasternal intercostal and intercostal muscles) and the soleus muscle. Although a trend existed, ICU care did not result in the significant atrophy of the type I fibers in the plantaris muscle. Note that 12 h of simulated ICU care did not promote the significant atrophy of type II fibers in any muscle investigated (Figure 8).

## 4. Discussion

ICU-acquired muscle weakness is a common complication of ICU care, and importantly, ICU-acquired weakness is associated with increased morbidity and mortality [18]. Currently, limited information exists about the mechanisms responsible for ICU-acquired weakness, and therefore, no standard preventive treatment for ICU-acquired weakness exists. This preclinical investigation provides the first evidence that simulated ICU care, independent of acute or chronic disease, results in a rapid disturbance in muscle proteostasis in both locomotor and accessory respiratory muscles. Indeed, as few as 12 h of simulated ICU care results in decreased anabolic signaling and protein synthesis in both the limb and respiratory muscles. Moreover, simulated ICU care rapidly activates muscle proteases in the limb and respiratory muscles. The next segments provide a critical analysis of the experimental model used in these experiments and discuss the significance of the new findings contained in this study. Importantly, we also examine the potential mechanisms contributing to ICU-induced disturbances in proteostasis within locomotor and respiratory muscles.

### 4.1. Critique of Experimental Model 

To investigate the impact of ICU care on skeletal muscle proteostasis, we used an established preclinical model of ICU care that incorporates controlled mechanical ventilation. The rat was selected as the experimental model because rat and human skeletal muscles share common functional characteristics, similar muscle fiber types, and exhibit a parallel time course in the development of ventilator-induced diaphragm atrophy [19,20,21]. Young adult female rats were studied because they maintain a relatively constant body weight from 4–6 months of age, and notably, no gender differences exist in VIDD between female and male rats [21,22]. Importantly, we have previously demonstrated that the skeletal muscle contractile dysfunction associated with prolonged muscle inactivity is not due to the use of sodium pentobarbital anesthesia [23].

A significant confounding variable that complicates the interpretation of human studies of ICU-induced muscle weakness is the presence of acute or chronic diseases. Indeed, both acute diseases (e.g., sepsis) and chronic diseases (e.g., cancer) can promote rapid muscle atrophy independent of muscle inactivity during ICU care. To avoid this, these experiments incorporated healthy adult rats. Therefore, the observed changes in skeletal muscle proteostasis within this investigation resulted from the inactivity associated with ICU care and not confounding variables that arise from diseases that promote muscle wasting (e.g., sepsis and/or cancer).

The impact of prolonged MV on the diaphragm has received considerable attention during the past two decades. In contrast, few studies have considered other important accessory muscles of ventilation. Therefore, this study explored the impact of prolonged MV on proteostasis within both the parasternal intercostals and the intercostal muscles. In rodents, the parasternal intercostals are inspiratory muscles that are active during quiet breathing. The intercostal muscles include both the internal and external intercostals; the external intercostals are inspiratory muscles, whereas the internal intercostals are active during expiration. In reference to the limb muscles, we studied both the plantaris and soleus muscles. These muscles were selected because both are active during locomotion and because these muscles differ markedly in fiber type composition. Specifically, the rat soleus muscle is primarily comprised slow, type I fibers (84% of total fiber type), whereas the plantaris muscle is dominated by fast, type IIx and IIb fibers (IIx + IIb = 80% of total fibers) [24]. This experimental approach provides the unique opportunity to determine if ICU-induced muscle wasting occurs preferentially in slow or fast muscle types.

Finally, to study the impact of MV on accessory respiratory muscles, our experiments incorporated controlled MV. During controlled MV, the ventilator provided full support for breathing, resulting in the complete inactivity of inspiratory muscles [20]. This experimental approach provides the opportunity to investigate the impact of respiratory muscle inactivity on proteostasis during simulated ICU care. 

### 4.2. ICU Care-Induced Impairment in Muscle Proteolysis Occurs Rapidly in Limb Skeletal Muscles

ICU-acquired weakness is a common clinical condition identified by skeletal myopathy with or without neurological impairment [25]. Indeed, limb muscle atrophy and weakness often develop during critical illnesses and are commonly diagnosed in patients requiring respiratory support (i.e., MV) during ICU care [26]. Specifically, limb muscle weakness has been reported in 26–65% of all ICU patients that were mechanically ventilated for 5–7 days [27,28]. Locomotor muscle wasting in ICU patients is reported to occur during the first 1–7 days of critical illness due to both a reduction in protein synthesis and increased protein breakdown [29,30,31]. For example, a clinical study involving ICU patients disclosed that muscle protein synthesis decreases and protein breakdown increases in the vastus lateralis muscle following 24 h of ICU care [31]. The current preclinical experiments support these findings and provide new details about the mechanisms responsible for ICU-induced skeletal muscle wasting. Indeed, the results of the current study are important for several reasons. First, to avoid the confounding variables of age and divergent diseases that are common to human studies of ICU-acquired weakness, the present preclinical experiments avoided this pitfall by studying healthy, young adult animals. Second, by investigating rodent limb muscles that differ in fiber type, this study provides new information about the effects that ICU care has on both slow and fast muscle fiber types. This contrasts human ICU studies in which the most studied limb muscle is the vastus lateralis; this muscle contains a mixture of fiber types (i.e., ~40% type I, 32% type IIa, and 20% type IIx) [32]. It follows that studies using the vastus lateralis do not provide insight into the impact of ICU care on different muscle fiber types. Finally, the present experiments also provide new information about the mechanisms responsible for ICU-mediated muscle wasting in both limb and respiratory muscles.

Our results are the first to reveal that as few as 12 h of simulated ICU care results in significant atrophy in type I (slow) fibers in the rat soleus muscle (Figure 8). Furthermore, although a trend toward atrophy also existed in type I fibers in the plantaris muscle during ICU care, this reduction in mean fiber CSA did not reach significance. To investigate the mechanisms responsible for ICU-acquired disturbances in proteostasis in limb muscles, we measured key biomarkers of anabolic signaling, the rate of muscle protein synthesis, and protease activity. Our results reveal that, compared to the control, ICU care promotes a rapid and significant decrease in muscle protein synthesis in both the plantaris muscle (i.e., ~−30%) and the soleus muscle (i.e., ~−40%) (Figure 3). Further, our findings indicate that simulated ICU care results in significant decreases in the levels of phosphorylated AKT, mTOR, and 4E-BP1 in both the soleus and plantaris muscles. Collectively, these novel findings indicate that ICU care results in the rapid depression of both anabolic signaling and protein synthesis in both types I and type II muscle fibers.

Our data also provide original information about the impact of ICU care on protease activation in both slow and fast muscle fibers. Specifically, 12 h of simulated ICU care did not activate calpain in either slow (soleus) or fast (plantaris) muscle fibers. In contrast, ICU care promoted the activation of caspase-3 in slow, type I fibers (soleus muscle) (Figure 4). Moreover, biomarkers of both autophagy and the ubiquitin-proteasome system were activated rapidly during simulated ICU care in both the soleus and plantaris muscles; this observation reveals that these proteolytic systems were active in both type I and type II muscle fibers (Figure 5 and Figure 6). Importantly, 12 h of simulated ICU care resulted in significant increases in phosphorylated STAT3 in both slow and fast muscle fibers (soleus and plantaris) (Figure 7).

Together, our findings indicate that as few as 12 h of simulated ICU care promotes limb skeletal muscle atrophy in slow muscle fibers. The mechanism(s) to explain this rapid atrophy in type I fibers appears to be due to the combination of accelerated proteolysis and depressed protein synthesis. Indeed, compared to the fast fiber type plantaris muscle, ICU care resulted in a slightly greater decrease in muscle protein synthesis and the greater activation of proteases (i.e., caspase-3 activation) in the slow soleus muscle. Although 12 h of ICU care is not sufficient to promote significant atrophy in type II muscle fibers, a relatively short period of ICU care does depress protein synthesis/anabolic signaling and increase proteolysis in fast muscle fibers; hence, it is predicted that muscle atrophy of type II fibers would occur with longer durations of ICU care.

### 4.3. ICU Care-Induced Impairment in Muscle Proteolysis Occurs Rapidly in Respiratory Muscles

In addition to providing unique information on the impact of ICU on limb muscles, our experiments also provide new information about the influence of simulated ICU care and prolonged MV on accessory muscles of ventilation. While numerous studies have probed the impact of prolonged MV on the principal muscle of inspiration (i.e., diaphragm), the current experiments provide new and important information about the effect that MV has on proteostasis in two key accessory muscles of respiration (parasternal intercostals and intercostal muscles). The parasternal intercostal muscles are inspiratory muscles and are active during quiet breathing in humans and other mammals [33]. In the rat, the parasternal intercostals are primarily comprised fast muscle fiber types (e.g., 4% type I fibers, 9% type IIa, 39% type Iix, and 48% type Iib) [34]. Note that the separation of the intercostal muscles into the internal and external intercostals is technically challenging, and inadvertently, the separation of these muscles often results in samples containing both muscles. Therefore, to avoid this problem, we investigated the intercostal muscles as an intact unit. As noted earlier, the external intercostal muscles are inspiratory muscles primarily composed of fast muscle fibers (4% type I fibers, 7% type Iia, 37% type Iix, and 52% type Iib) [33,34]. The internal intercostal muscles act as expiratory muscles and are identical to the external intercostals the internal intercostals are dominated by fast muscle fiber types (e.g., 4% type I fibers, 7% type IIa, 37% type IIx, and 52% type IIb) [33,34]. Our results reveal that, in both the parasternal intercostals and the intercostal muscles, ICU care combined with MV results in large decreases in anabolic signaling and muscle protein synthesis (Figure 2 and Figure 3). Moreover, ICU care in combination with MV results in significant increases in the expression of muscle-specific E3 ligases (MuRF1 and MAFbx/Atrogin-1) and the activation of autophagy (an increased ratio of LC3II/I) in both these respiratory muscles (Figure 5 and Figure 6). Additionally, ICU care combined with MV increased the phosphorylation of STAT3 for both parasternal and intercostal muscles (Figure 7). Importantly, the combination of this depressed protein synthesis and accelerated proteolysis resulted in the rapid and significant atrophy of type I muscle fibers in both the parasternal intercostals and the intercostal muscles (Figure 8). To our knowledge, these data provide the first evidence that as few as 12 h of MV results in accelerated proteolysis, depressed protein synthesis, and anabolic signaling, along with the atrophy of type I muscle fibers in the accessory muscles of respiration. 

## 5. Summary and Conclusions

Our results support the hypothesis that ICU-induced muscle inactivity results in a rapid decline in both anabolic signaling/protein synthesis and accelerated proteolysis in both limb and respiratory muscles. Indeed, our findings provide the first evidence that disturbances in proteostasis occur in both locomotor and respiratory muscles within the first 12 h of a simulated ICU environment that includes ventilator support. Importantly, these ICU-induced disturbances in skeletal muscle proteostasis occur irrespective of muscle function or muscle fiber type.

While the consequences to quality of life and long-term outcomes of skeletal muscle wasting in critical care patients are recognized, no therapeutic interventions are currently available to prevent the detrimental effects of prolonged immobilization in the ICU. A precise understanding of the mechanisms leading to ICU-induced weakness is essential to developing therapeutic treatments to prevent disturbances in proteostasis during critical illness. In this regard, our experiments provide new evidence that decreases in both anabolic signaling and protein synthesis occur in limb and respiratory muscles within the first 12 h of simulated ICU care. Further, our data reveal that key proteolytic systems (i.e., caspase-3, ubiquitin-proteasome, and autophagy) are rapidly activated in limb and respiratory muscles during ICU care. These novel findings provide a foundation for developing therapeutic treatments to prevent the decreases in anabolic signaling/protein synthesis and the acceleration of proteolysis in skeletal muscles during ICU care.

## Data Availability

Individual data points are plotted within the figures contained in this publication.
